# Gaining Insights from *Candida* Biofilm Heterogeneity: One Size Does Not Fit All

**DOI:** 10.3390/jof4010012

**Published:** 2018-01-15

**Authors:** Ryan Kean, Christopher Delaney, Ranjith Rajendran, Leighann Sherry, Rebecca Metcalfe, Rachael Thomas, William McLean, Craig Williams, Gordon Ramage

**Affiliations:** 1 School of Medicine, Dentistry and Nursing, College of Medical, Veterinary and Life Sciences, Glasgow G2 3JZ, UK; ryan.kean@uws.ac.uk (R.K.); c.delaney.1@research.gla.ac.uk (C.D.); ranjith.rajendran@glasgow.ac.uk (R.R.); leighann.sherry@glasgow.ac.uk (L.S.); William.McLean@glasgow.ac.uk (W.M.); 2 Institute of Healthcare Policy and Practice, School of Health, Nursing, and Midwifery, University of the West of Scotland, Paisley PA1 2BE, UK; craig.williams@uws.ac.uk; 3 Sandyford Sexual Health Service, NHS Greater Glasgow and Clyde, Glasgow G3 7NB, UK; rebeccametcalfe@nhs.net (R.M.); rachaelthomas2@nhs.net (R.T.)

**Keywords:** *Candida*, biofilm, antifungal

## Abstract

Despite their clinical significance and substantial human health burden, fungal infections remain relatively under-appreciated. The widespread overuse of antibiotics and the increasing requirement for indwelling medical devices provides an opportunistic potential for the overgrowth and colonization of pathogenic *Candida* species on both biological and inert substrates. Indeed, it is now widely recognized that biofilms are a highly important part of their virulence repertoire. *Candida albicans* is regarded as the primary fungal biofilm forming species, yet there is also increasing interest and growing body of evidence for non-*Candida albicans* species (NCAS) biofilms, and interkingdom biofilm interactions. *C. albicans* biofilms are heterogeneous structures by definition, existing as three-dimensional populations of yeast, pseudo-hyphae, and hyphae, embedded within a self-produced extracellular matrix. Classical molecular approaches, driven by extensive studies of laboratory strains and mutants, have enhanced our knowledge and understanding of how these complex communities develop, thrive, and cause host-mediated damage. Yet our clinical observations tell a different story, with differential patient responses potentially due to inherent biological heterogeneity from specific clinical isolates associated with their infections. This review explores some of the recent advances made in an attempt to explore the importance of working with clinical isolates, and what this has taught us.

## 1. What Is Biofilm Heterogeneity?

**Figure 1:**
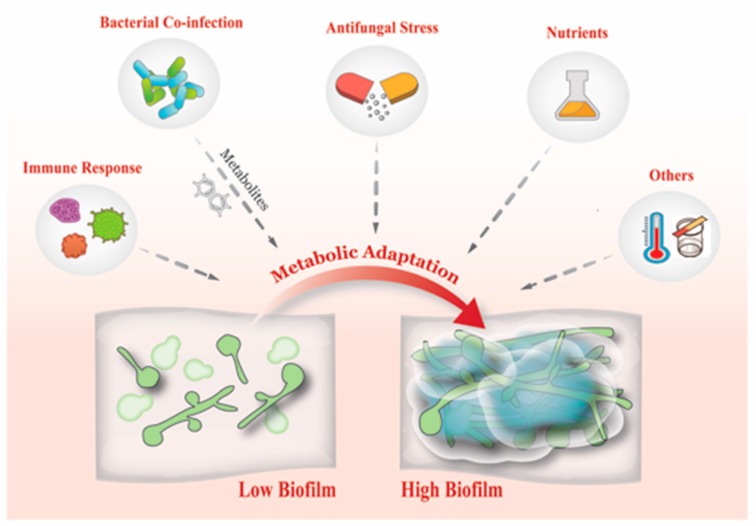
Factors influencing biofilm formation. There are multiple stimuli that can induce biofilm formation including the immune response, antifungal stress, and bacterial derived metabolites. Environmental stressors can also stimulate biofilm formation, and these include the availability of nutrients, temperature, and pH. Dashed arrows represent different factors that associate with biofilm formation.

Classical molecular microbiological approaches suggest that deletion or over expression of particular genes enables us to definitively deduce their function. Reinforced by structural biology studies, these tactics allow us to deduce the structure/function of particular proteins within the context of a microbes pathogenic ability. Nevertheless, this assumes that molecular manipulations do not have any pleiotropic effects, nor does this take into account inherent biological heterogeneity that bears itself amongst a range of clinical isolates (Figure 1). This begs the question whether using laboratory strains is the optimal way in developing our understanding of microbial pathogenesis [1], or instead, whether taking a combinatory approach through evaluating phenotypic and genotypic characteristics of clinical isolates would enhance our understanding. This review focuses on *Candida* biofilms and attempts to examine the literature with respect to what insights can be garnered from working with clinical isolates and observing the inherent heterogeneity that exists.

## 2. How Do We Investigate Biofilm Formation?

The key driver in understanding and evaluating biofilm formation from important *Candida* species lies in the quantitative methods utilized. When screening large collections of clinical isolates from different patient cohorts, several experimental strategies have been utilized, predominantly quantifying biomass using dry weight, stains such as crystal violet, and the metabolic dye XTT [2]. Each technique has their own benefits and caveats, but caution must be taken when interpreting the data achieved from each assay, particularly when correlating it to clinical outcomes. Given the heterogeneity found between strains, alongside varying laboratory models and techniques, standardization becomes problematic. For example, two of the most commonly used media for biofilm formation are Roswell Park Memorial Institute (RPMI) media and Spider media. Studies have identified that RPMI is more supportive of biofilm formation, stimulating biofilms that are three times thicker than Spider media [3]. Furthermore, these media are not physiologically relevant, with several studies employing more biologically relevant conditions for biofilm formation through use of artificial saliva, urine, and mammalian serum [4,5,6]. One of the most commonly used bioassays is the sodium salt XTT (2,3-bis(2-methoxy-4-nitro-5-sulfo-phenyl)-2*H*-tetrazolium-5-carboxanilide) [7,8]. This biofilm assay is highly reproducible and allows for a high throughput of multiple microtiter plates without compromising accuracy. Its usefulness comes with susceptibility testing, allowing for the direct comparison of antifungal treated samples compared to an untreated control [9]. Given the metabolic variation observed between both different strains and species, caution must be taken when interpreting the assay, as a measurement for biofilm development may simply be a reflection of high cell numbers [10,11]. For example, scant biofilms of non-albicans yeasts may show a high XTT value, yet minimal biomass is present. Therefore, the output achieved from XTT is only cellular viability and it does not take into account other biofilm components such as the extracellular matrix (ECM), which are arguably the most important when it comes to biofilms [12].

Another commonly used assay for biofilm formation is crystal violet staining. This method provides the total quantification of the biofilm biomass (cells and ECM) and also allows for rapid, high-throughput processing of multiple samples. However, variability of the washing step can result in both over- and under-estimation of biomass, with the assay also unable to differentiate subtle differences between samples [2]. An interesting example of this was described in a recent study, where these techniques were used to stratify the ability of *Candida* bloodstream isolates to form biofilms [13]. There was no evident standard for their stratification to denote strains as biofilm or non-biofilm formers, with a crystal violet values of OD_570_ > 0.09 simply denoted as a biofilm former. By doing so, it was concluded that non-*Candida albicans* species (NCAS) form greater biofilms than *C. albicans*, and that biofilm formation does not correlate to clinical outcomes. This is contrary to a wealth of previous literature, whereby the ability of *Candida* isolates to form a biofilm does associate with mortality [14,15,16,17].

Discrepancies between these findings illustrates the necessity for standardised testing to elucidate biofilm-related risk factors. Our group has taken a “belt and braces” approach, using a combinational approach of crystal violet, XTT, and SYTO^®^9 fluorescence quantitative biofilm assays (Thermo Fisher Scientific, Paisley, UK). Here, significant correlations were observed for *C. albicans* biofilm formation, which was subsequently used to stratify biofilm-forming ability [14]. Irrespective of the particular quantitative approach, wide-spread biofilm heterogeneity is observed within different clinical panels of isolates [13,14,18,19]. Collectively, these data suggest that different *Candida* strains function differently, and that consideration should be given to the individual isolates as we try and understand their clinical importance with respect to antifungal resistance and pathogenic potential.

## 3. Is Heterogeneity Clinically Important?

Since the earliest descriptions of *Candida* biofilms, great strides have been made to unequivocally demonstrate their clinical significance, despite perceived contention in the field. Throughout the human host, *Candida* biofilms colonize a wide variety of anatomical locations, as shown in Table 1. The oral and vaginal epithelium provide a mucosal niche for biofilm formation, whilst indwelling medical devices such as prosthetic heart valves and central venous catheters provide an inert, abiotic substrate for subsequent biofilm adherence and proliferation [20,21]. Irrespective of isolation site, biofilm heterogeneity has been reported, including the oral cavity, bloodstream, and urinary tract [14,22,23,24,25,26].

Within a clinical setting, intravascular catheters provide an optimal environment for *Candida* spp., allowing for the development and maturation of biofilms to which cells can disperse and subsequently cause candidaemia. Dispersed biofilm cells have been shown to be more pathogenic than their planktonic counterparts, exhibiting greater cytotoxicity and virulence in vivo [50]. Therefore, the role of the biofilm phenotype has potentially profound implications within the clinical environment. An initial study from Tumbarello and colleagues (2007) [17] aimed to identify the top risk factors associated with mortality rates in candidaemia patients. Using multivariate analysis, they were able to distinguish inadequate antifungal therapy (odds ratio (OR) 2.36, *p* = 0.03), APACHE III (OR 1.03, *p* < 0.001), and overall biofilm-forming *Candida* species (OR 2.33, *p* < 0.007) as significant variables associated with mortality [17]. When scrutinized at the *Candida* species level, only *C. albicans* (OR 3.97, *p* < 0.001) and *C. parapsilosis* (OR 4.16, *p* = 0.03) were shown to significantly correlate to biofilm-based mortality. A follow up study subsequently identified that central venous and urinary catheters, use of total parenteral nutrition, and diabetes mellitus as independent entities of bloodstream infections caused by biofilm forming isolates [16]. Furthermore, they demonstrated the potential economic burden of these isolates resulting from increased lengths of hospital stays and use of antifungals and ultimately resulted in an increased possibility of mortality [16]. A more recent, prospective analysis subsequently identified line removal (*p* = 0.032) as a significant risk factor associated with mortality rates from a candidaemia patient cohort, with the removal of an indwelling line correlating with a more positive patient outcome [14]. Interestingly, when this was then subsequently assessed at *Candida* species level, survival analysis demonstrated significantly higher survival rates for patients with *C. albicans* associated line removal compared to no removal, with no differences observed in NCAS [51]. Furthermore, Tascini and colleagues used a random forest model of analysis to cluster candidaemia associated mortality and to identify its accompanying risk factors [52]. It was shown that azole use and high APACHE II, as well as biofilm formation, significantly correlated with the highest mortality group [52]. Published guidelines have suggested that catheter-related bloodstream infections should result in the direct removal of such devices, if possible [53,54,55]. Furthermore, a meta-analysis of seven clinical trials revealed that the removal of central venous catheters significantly correlated with reduced mortality rates (OR 0.50, *p <* 0.001) [56]. Conversely, a study assessed the efficacy of catheter removal within 24 h to 48 h of antifungal therapy and demonstrated no clinical improvement. This study, however, looked at echinocandins and liposomal amphotericin B, two highly active *Candida* biofilm agents [57]. What these studies do provide is an insight into differential responses to biofilm-active therapies, and suggest clinical isolates respond differently depending on their capacities to form biofilms. Despite the majority of studies focusing on the potential for *Candida* biofilms to develop on hard, abiotic surfaces, there are a variety of mucosal niches within the host to which *Candida* can colonise as a biofilm an induce tissue damage.

Key to successful colonisation and host damage to a mucosal niche is the secretion of various hydrolytic enzymes. These secreted proteins are a primary attribute within the virulence armamentarium of the organism allowing it to invade host tissue, and include proteinases, haemolysins, and phospholipase. Of these enzymes, the secreted aspartyl proteinases (Saps) are the most studied, comprising a family of ten genes (*SAP1–10*). The secretion of these enzymes has been attributed with disease, with high levels of expression observed from a variety of diseases including infections of the bloodstream, vagina, oral cavity, and diabetes mellitus [58,59,60]. Given the diversity of the Sap family, then differential expression of independent genes has been associated with varying anatomical location [59,61]. During biofilm formation, *SAP5* is up-regulated, significantly correlating with biomass [19]. Indeed, an integrated global substrate and proteomics approach identified *SAP5* and *SAP6* as the major biofilm-related proteases utilised by *C. albicans*. Manipulation of both of these genes resulted in decreased adhesion and impaired biofilm development both in vitro and in vivo, highlighting their role as potential biofilm biomarkers [62]. Recent studies have identified a novel fungal toxin termed candidalysin, a hyphae-specific peptide critical for epithelial damage [63] and expression of the gene coding this toxin (*ECE1*) was shown to be highly up-regulated in *C. albicans* isolates capable of forming biofilms [64].

An area worthy of consideration for mucosal biofilm formation is vulvovaginal candidiasis (VVC). Although not life-threatening per se, this infection will affect up to 75% of women in their lives at least once and are one of the most common fungal infections globally [65]. While the majority of these cases are sporadic and will clear after one episode, some women will emerge with persistent occurrences (>4 episodes a year), despite being completely asymptomatic between these episodes (recurrent VVC (RVVC)) [66]. The reasoning for RVVC is multi-factorial, yet given that azoles are first line topical drug of choice and have widespread availability from over the counter, then inadequate therapy is extremely problematic. While biofilm formation is regarded as a pathogenic attribute of bacterial vaginosis, its role in RVVC remains equivocal, despite a growing body of evidence to suggest otherwise [23,67,68,69]. *Candida* biofilms have been shown to form on the vaginal mucosa in vivo, as well as on inert substrates such as intrauterine contraceptive devices [67,70]. A recent study from our group screened a cohort of 300 VVC isolates for their epidemiology, biofilm formation, and azole susceptibility [23]. Interestingly, an epidemiological shift towards NCAS was observed, and that biofilm formation was heterogeneous between these isolates regardless of *Candida* species. For *C. albicans*, it was demonstrated that the planktonic MIC_50_ for fluconazole was 4 mg/L, yet when the susceptibility profile of these isolates was tested as biofilms, the MIC_50_ escalated to >32 mg/L. This highlights the role for the biofilm phenotype, and may go towards explaining the chronic phenotype in this patient cohort and irresponsiveness to treatment.

## 4. How Does Heterogeneity Impact Antifungal Treatment?

Antifungal resistance is a complex, multifactorial process to which can be induced in response to a compound or as an irreversible genetic alteration as a result of prolonged drug exposure. While resistant planktonic cells predominantly arise from inherited traits to maintain a resistant phenotype, biofilm resistance rises through mechanisms such as over-expression of target molecules, efflux pump activity, and through the protective barrier of the extracellular matrix (ECM) allowing limited diffusion. Undoubtedly, the most defining characteristic of biofilms is this intrinsic and adaptive recalcitrance to many antimicrobial therapies. Compared to their free-floating planktonic equivalents, up to 1000-fold higher concentrations of antifungal agents can be required to effectively kill *Candida* biofilms in vitro, with the same decreased sensitivities also observed in vivo [71,72].

**Figure 2:**
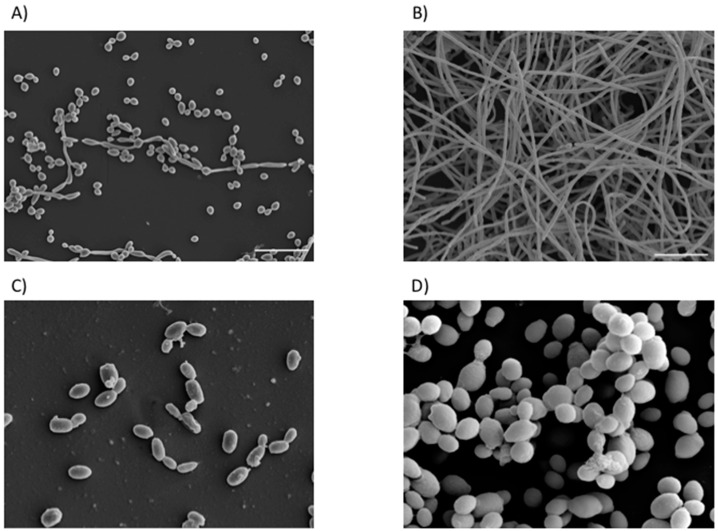
Differential biofilm formation of *Candida* species. Scanning electron micrograph (SEM) of *C. albicans* low biofilm formers (LBF) (**A**) existing as mainly yeast cells and pseudo-hyphae, compared to the hyper-filamentous morphology of the high biofilm formers (HBF) (**B**); Micrograph of *C. glabrata* biofilm sparsely populating the surface (**C**); SEM image of a biofilm formed by an aggregating strain of *C. auris* (**D**). Scale bars represent 20 µm at ×1000 magnification.

Several clinical observations have associated the ability to form biofilms with mortality, but also with azole and inadequate antifungal use. Many studies have sub-categorised *C. albicans* isolates as low biofilm formers (LBF) and high biofilm formers (HBF) [14,73,74]. Phenotypically, biofilms formed by these isolates are distinct, with LBF existing predominantly as sparse populations of yeast cells and pseudohyphae, whereas HBF have a dense, tenacious hyphae based morphology (Figure 2). In vivo, there is also biological differences, with increased mortality rates observed in HBF compared to LBF [19,75]. Additionally, it was shown in vitro that isolates categorised as LBF and HBF were differentially sensitive to azoles and echinocandins at both low and high dosage, with the later less susceptible to these concentrations [14]. Furthermore, HBF are less responsive to amphotericin B therapy, with an eight-fold increase in concentration needed to achieve an 80% kill in this population [19].

Prolonged and inadequate antifungal exposure has resulted in the emergence of both azole and echinocandin resistant strains [76,77]. Partly responsible for this problem is the occurrence of heteroresistance (HR), a phenomenon described in both prokaryotic and eukaryotic pathogens [78,79,80]. HR is defined as sub-populations of cells existing within a primarily susceptible population, able to survive antimicrobial challenge higher than the strains given minimum inhibitory concentration. These cells are unidentifiable from within this population using standard broth microdilution assays [81]. Furthermore, it has repercussions of inadequate antifungal therapy, resulting in ineffective patient management and potential chronic infection. The potential role of HR in biofilms remains unclear, yet given that HR has been shown to significantly correlate with the upregulation of efflux membrane transporters [78], a key azole resistance mechanism of *Candida* biofilms [82,83], then it is highly plausible that they may occur within biofilm communities.

Another theory that yields to heterogeneous drug tolerant sub-populations are persister cells. While they have been extensively characterised within bacterial pathogens [84], their role in *Candida* is less well defined. Persister cells are described as a population of dormant cells, heterogeneously populated throughout a biofilm that remain tolerant to antimicrobial therapy [84]. Despite demonstrating similar recalcitrant phenotypes, there remains fundamental differences between persister and HR cells. Persister cells display phenotypic differences yet remain genetically identical to the remainder of their population [85]; this is unlike HR, whereby genetic alteration as a consequence of aneuploidy may potentially play a role in these drug tolerant phenotypes. However, it has been demonstrated that diploid biofilms contain 10 times the number of persister cells within a population, compared to haploid biofilms. Interestingly, overexpression of *AHP1* in the haploid genotype restored the persister cell population [86]. Furthermore, the active mechanism of persister cells remains unknown, yet has been shown not to be linked to efflux pumps [87], whereas HR levels have been shown to correlate to efflux pump activity [78]. In *Saccharomyces cerevisiae,* the TORC1 pathway is involved in promoting amphotericin B persistence, with inhibition of this pathway also increasing the quantity of persisters in *C. albicans* and *C. glabrata* [88]. A proteomics approach was taken to characterise *C. albicans* biofilm persister cells following exposure to high amphotericin B treatment [89]. These cells were shown to have a unique profile, displaying 205 differently expressed proteins. Interestingly, the up-regulation of the stress response from the heat shock protein (HSP) family was identified, including HSP90, a key regulator of biofilm dispersion and drug resistance [90]. While there have only been a limited number of studies in fungal biofilms with respect to peristers, predominantly focusing on *C. albicans*, they are also studies in a number of NCAS, including *C. krusei, C. parapsilosis*, and *C. glabrata* [88,91,92].

## 5. Do Non-Albicans Species Play a Role?

Despite *C. albicans* being regarded as the principal biofilm forming pathogen of the genus, there has been a steady flow of research looking at non-albicans biofilms over the last decade. A recent study from Soldini and colleagues (2017) [15], demonstrated biofilm heterogeneity within a *C. parapsilosis* candidaemia patient group. By grouping these isolates into HBF and LBF, they identified that central venous catheter (CVC)-related candidaemia and a poorer patient outcome were significantly associated with the HBF group [15]. The clinical consequences of NCAS have also been described with *C. glabrata* being identified frequently from catheter-associated candidaemia [93]. A study from Silva and colleagues (2009) [94] assessed the biofilm forming ability and matrix composition of a panel of NCAS isolates. They showed that *C. glabrata* demonstrated low levels of biofilm formation, whereby little heterogeneity was observed in terms of biomass. These observations may be explained by the lack of sensitivity in the biomass assays, being unable to detect subtle differences low-level biofilms. In addition, both *C. parapsilosis* and *C. tropicalis* displayed generally greater biomass, with heterogeneity observed between the isolates they tested [94]. Interestingly, *C. parapsilosis* and *C. glabrata* biofilm ECM was predominantly composed of carbohydrate compared to *C. tropicalis*, to which had both low carbohydrate and protein content [94]. The dynamics of multi-*Candida* spp. biofilms in response to antifungal agents have been evaluated, showing that in the presence of other species, *C. albicans* lost its compositional dominance within the biofilm in response to antifungal treatment, and that *C. glabrata* and *C. tropicalis* demonstrated reduced susceptibility to amphotericin B when in the mixed-species biofilm [95]. Additionally, it has been shown that *C. albicans* can augment the virulence of *C. glabrata* with regards to its invasive capacity. As seen with numerous species of bacteria, *C. glabrata* preferentially binds to the hyphal elements of *C. albicans*, thus enhancing its invasion of oral tissues, analogous to injection from a needle stick [96,97].

Of recent interest in the medical mycology field is the emerging pathogen *Candida auris*. It has received considerable attention due to its resistance profile, difficulty for accurate identification, and its ability to cause hospital outbreaks. It is phylogenetically similar to *C. lusitaniae* and *C. haemulonii*, yet there is noticeable differences compared to many other *Candida* spp. [98]. There has been a simultaneous emergence of distinct clades of this organism in different geographical locations, currently categorised into the East Asian, South Asian, South American, and South African clades [99,100]. Between these four clades, there is extensive genetic variance, yet minimal internal clade differences [99]. In vivo it is highly virulent, with invertebrate models demonstrating comparable virulence to *C. albicans* [37,101]. Initial studies identified two distinct phenotypes between clinical isolates, existing as either aggregates or single cellular communities. Whilst the former appears to be generally differentially susceptible to azoles, the latter is significantly more virulent, likely due to an inability of aggregative strains to cause disseminated infection [37]. The ability of *C. auris* to form biofilms was initially disregarded [102], reporting no ability to form biofilms, although the semi-quantitative methods used here were rudimentary. In fact, *C. auris* is able to form biofilms as recently demonstrated by Sherry and colleagues (2017) [103], whereby they showed that it was able to form intermediate levels of biomass compared to *C. albicans* and *C. glabrata*. Although these biofilms were not comparable to *C. albicans*, they demonstrated a highly resistant susceptibility profile across all classes of antifungals, most notably to echinocandins and polyenes, whereby they up to 256- and 16-fold increases in MIC against micafungin and amphotericin B were observed, two antifungal agents usually potent against *C. albicans* biofilms [103]. Further studies have identified that the glucan synthase inhibitor SCY-078 possesses activity against *C. auris* biofilms, reducing the biofilm thickness and viability, these studies did however use a 48-h treatment regimen [104]. Given the multi-drug resistant phenotype of this organism, then its control within the nosocomial environment is imperative. It has been shown to successfully colonise the skin [45], as well as successfully persist on plastics and steel for prolonged periods [105,106]. Furthermore, disinfection procedures have has variable outcomes [107], with increased concentrations and exposure times of disinfectants required to successfully eliminate the organism [108].

## 6. Interkingdom Interactions Support Biofilm Defects

Due to advances in the use of more sophisticated biofilm techniques, it is now widely appreciated that in addition to their own clinical biofilm heterogeneity, these biofilms rarely exist as single entities. In fact, they often exist as complex, diverse, and heterogeneous cellular communities of organisms spanning different phylogenetic kingdoms [109]. Interactions through both a physical and chemical nature can have negative implications for human health through the production of intensified pathogenic phenotypes and increased tolerance to antimicrobial challenge. *C. albicans* is the most common fungal pathogen frequently co-isolated from polymicrobial biofilm infections and can interact with a number of different bacteria in a variety of ways (Table 1). While research within this field has intensified in recent years, the majority of studies are tailored to the use of characterised laboratory strains, with minimal focus on the impact of these interactions within clinical isolates. A study from O’Donnell et al. (2017), observed that the increasing *Candida* load promoted an altered microbiome of denture wearers [39]. The highest correlation was observed with *Lactobacillus* species, whereby increased *Candida* burden resulted in higher abundance of *Lactobacillus*. This suggestive positive relationship is conflicting to previous literature whereby an antagonistic interaction was observed [110]. Collectively this study highlights potential novel avenues of research into understanding interkingdom interactions through the use of clinical isolates.

Chronic infections, including burn wounds and diabetic foot ulcers (DFU), are of an increasing interest due to their economic burden and substantial contribution of morbidity and mortality [111]. These chronic infections often comprise pathogenic, polymicrobial biofilms, thus complicating treatment regimens [112,113,114]. Associated with these treatment complications are the underappreciation of fungi as major facilitators within these communities [115]. It was recently demonstrated in a newly developed triadic biofilm model containing *C. albicans*, *Pseudomonas aeruginosa*, and *Staphylococcus aureus* [114], that single antimicrobial treatments of flucloxacillin and ciprofloxacin were ineffective due to the fungal contingent within the biofilm [116]. Indeed, it was shown that, in order to achieve substantial reduction in overall bioburden, an antimicrobial cocktail containing both fungal and bacterial specific agents needed to be applied. Biofilms containing these organisms have also been shown to be recalcitrant to disinfectant strategies, with polymicrobial biofilms showing decreased susceptibility as compared to their single-species equivalents [117]. Collectively, these findings highlight not only the need for accurate antifungal approaches to *Candida* biofilm infections but also the appreciation of the universal fungal influence towards treatments for polymicrobial infections.

**Figure 3:**
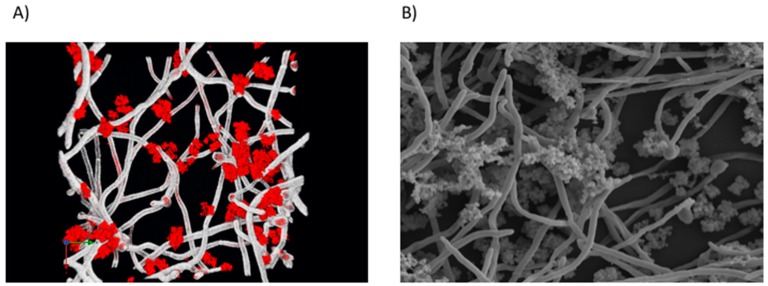
*Candida albicans* and *Staphylococcus aureus* dual-species biofilm. Confocal laser scanning micrograph (CLSM) (**A**) and scanning electron micrograph (SEM) (**B**) highlighting the close interaction between the bacteria (red) and fungal hyphae (white). The *C. albicans* mycofilm acts as a scaffold for *S. aureus* colonisation and biofilm formation. Images are viewed at ×2000 magnification.

Perhaps the most well-studied mechanism of *Candida*–bacteria interactions is physical attachment. A scaffold of hyphae within a biofilm provides a potential niche for the colonization of various Gram-positive and Gram-negative bacteria, a phenomenon we have termed as a ‘mycofilm’ (Figure 3) [6]. This term is proposed through the ability of *C. albicans* to promote biofilm formation of a normally biofilm defective strain of *S. aureus.* The bacteria preferentially adhere to hyphal as opposed to yeast cells [118], mediated by the *C. albicans* agglutinin-like sequence 3 protein (Als3p) [119], though it is likely that other reciprocal proteins are involved. Indeed, this bacterial attachment can also be reduced through enzymatic degradation of extracellular DNA (eDNA), while concomitantly increasing miconazole susceptibility to dual species biofilms [6]. The ECM is a defining characteristic of these biofilms, with initial studies highlighting the protective effect of *C. albicans* to *S. aureus* vancomycin therapy [120]. Further studies have now intricately identified fungal β-1,3-glucan as the matrix component to which drives this resistance [121]. The authors propose a ‘barrier model’ as the active mechanism of resistance, whereby as the biofilm matures, *S. aureus* becomes coated in the secreted fungal matrix constituents thus impeding the activity of vancomycin. Interestingly, this same component has been shown to promote fungal-derived ofloxacin tolerance in an *Escherichia coli* and *C. albicans* dual-species biofilm [122].

A less well-studied mechanism within polymicrobial biofilms is metabolic cross-talk between organisms. Chemically mediated signaling in the form of quorum sensing (QS) may play a potential role within these interactions, stimulating both positive and negative effects, potentially giving *C. albicans* a fitness advantage within polymicrobial niches. The *C. albicans* QS molecule farnesol has been shown to be an important regulatory molecule in biofilm formation [123], as well as being able to decrease bacterial biofilm formation and potentiate antimicrobial therapy [124,125]. Interestingly, although being able to inhibit *Strepotococcus mutans* biofilm formation at high concentrations (~200 µm), farnesol has been shown to promote biofilm formation and micro-colony development of *S. mutans* at lower concentrations (25–50 µm) [126]. Additionally, *S. mutans* is able to reduce the quantity of farnesol produced in these dual-species environment, suggesting that this molecule plays a key role in biofilm formation in oral plaque.

Throughout *Candida* biofilms, there is a heterogeneous oxygen gradient, which decreases steadily from the top to the bottom of the biofilm architecture. Interspersed within the dense biofilm network are hypoxic niches, creating small oxygen-deprived microenvironments. These micro-niches, have been shown to support the growth of various anaerobic bacteria [127,128]. Fox and colleagues [128] demonstrated that in the presence of biofilms containing *C. albicans*, various anaerobic gut microbiota bacteria were able to survive in normoxic conditions that were typically toxic to the bacteria. Reciprocally, they demonstrated that *Clostridium perfringens* was able to induce *Candida* biofilm formation and also phenotypic switching from white to the opaque cell type through upregulation of the transcriptional regulator *WOR*1. Another *Clostridia* species, *C. difficile*, has also been shown to survive within the hypoxic microenvironments within *C. albicans* biofilms, yet conversely inhibited hyphal formation. It instead induced the hyphae-yeast transition, through the molecule *p*-Cresol [127].

The diversity of the human microbiota coupled with the advancement in interest of the mycobiome provides a platform for endless opportunities for interaction of different microbes. Through comparison of the micro- and mycobiome in patients with Crohn’s disease (CD), it has been shown that specific fungal-bacterial interactions associate with dysbiosis in CD [129]. The authors then identified a correlation between the fungus *C. tropicalis* and the bacteria *Serratia marcescens* and *E. coli*. This was then validated in vitro whereby they demonstrated the ability of these three organisms to form triadic biofilms that promoted overall biomass and stimulated hyphal formation of *C. tropicalis.* Though caution should be exercised in reliance of statistical relationships, as these approaches lead to the exclusion of perceived bystander microorganisms that may have greater functional importance than are currently considered.

## 7. What Drives Biofilm Heterogeneity?

Through experimental advancement and use of more sophisticated technologies, *Candida* biofilm ECM has been extensively analysed [130,131,132,133]. Compositionally, the ECM is comprised of four main macromolecular constituents: proteins, carbohydrates, lipids, and nucleic acid. However, through use of a multi-omics approach, Zarnowksi et al. (2014) identified an abundance of novel components within these four subclasses, generating a distinguished compendium of its constituents. This demonstrated its clinical relevance of providing biofilm stability, sequestration of drugs, and protection from the surrounding environmental stressors, as well as subsequently facilitating biofilm dispersal [130]. While the majority of ECM-mediated research has focused on the role of polysaccharides, another notable component is eDNA [134]. Despite only contributing to 5% of the ECM, eDNA plays a substantial role in maintaining structural homeostasis within the matrix. It is thought to act as molecular glue, facilitating cohesion between the other matrix constituents. Exogenous addition and enzymatic depletion of eDNA have been shown to both positively and negatively influence biofilm formation, respectively [134]. Additionally, the addition of DNase to amphotericin B and caspofungin enhances their activity against sessile communities, however, no positive interaction is observed with azoles [135]. Interestingly, eDNA is also factor that contributes for the biofilm forming heterogeneity observed between LBF and HBF. Significantly increased quantities of eDNA were released from both early and mature biofilms of HBF compared to LBF [136]. Given that HBF are more resistant to amphotericin B (AMB) than LBF [19], the combination therapy with AMB and DNase, which sensitises HBF up to eight-fold compared to AMB alone, is very much a matrix-mediated resistance [136]. The role of the other ECM components within biofilm heterogeneity observed in clinical isolates remains unknown, yet given the differences observed between azole and echinocandin susceptibility of these isolates [14], it is highly likely that key components are involved and worthy of further scrutiny to determine if strain specific ECM motifs are present.

Given the complexity of the biofilm formation process, it is unsurprising that a variety of transcriptional regulations determine this process. Central to this is the master regulatory transcriptional network as defined by Nobile and collegues (2012) [137]. Originally, a hub of six regulatory genes (*TEC1*, *NDT80*, *ROB1*, *BRG1*, *BCR1*, *EFG1*) was identified that regulate both themselves and approximately 1000 genes involved in processes biofilm formation such as hyphal morphogenesis, ECM production, and drug resistance [137]. Furthermore, this same group then identified an additional three regulatory genes responding to temporal changes in biofilm formation. Using deletion strains, they identified FLO8 as a regulator throughout all stages of development, from initial adherence to fully mature biofilms, whereas RFX2 and GAL4 are required only in the later stages of maturation [138]. Interestingly, when comparative transcriptomes between *C. albicans* and *C. parapsilosis* biofilms were analysed, they contained a distinct variation between the two species. A large transcription factor deletion screening identified eight biofilm regulators in *C. parapsilosis*. Of the regulatory network of *C. parapsilosis*, only EFG1 and BCR1 are also involved within the categorised *C. albicans* network [139]. While these approaches provide invaluable insights into the transcriptional mechanisms underpinning biofilm development, their limitations lie within only considering laboratory reference strains. Indeed, when the transcriptional profile of a group of *C. albicans* LBF and HBF were compared, no transcriptional differences of two of the master biofilm regulators (BCR1 and EFG1) was shown, despite the phenotypic and biological differences between the strain subsets [19].

As well as a defined transcriptional network governing biofilm formation, various metabolic circuits control the transition from planktonic cells to biofilm maturity. Using a metabolomics approach, Zhu and colleagues (2013), performed a time-course analysis of the metabolome of *C. albicans* biofilms through development [140]. They identified 31 metabolites that were differently expressed between planktonic and biofilm cells that were involved in various processes, including the TCA cycle, amino acid biosynthesis, and oxidative stress. Interestingly, they showed that trehalose was highly up-regulated after 6 h of maturation. Using a *TPS1* knockout, they demonstrated an impaired biofilm phenotype, as well as increased sensitivity to amphotericin B and miconazole, thus highlighting the importance of the trehalose biosynthesis pathway for biofilm maturation [140].

**Figure 4:**
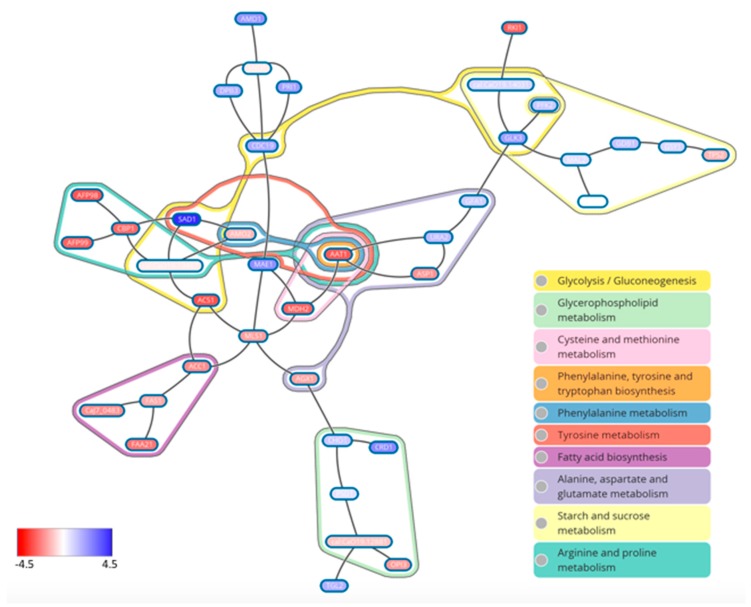
Maximum scoring metabolic subnetwork in the LBF-HBF network. Differential transcriptional expression between LBF and HBF. Red gene names indicate upregulation in HBF, with blue indicating LBF.

In order to better our understanding of the molecular mechanisms facilitating biofilm heterogeneity between *C. albicans* clinical isolates, Rajendran and colleagues (2016) undertook a transcriptional profiling approach [64]. As expected, well-known biofilm-related genes such as *HWP1* and *ALS3* were up-regulated in HBF. A non-biased computational approach was further utilized, and in doing so, a metabolic circuitry to defined biofilm phenotypes was established (Figure 4). Using KEGG pathway analysis, it was shown that the amino acid pathways arginine and proline metabolism, pyruvate metabolism, and also fatty acid metabolism, were highly expressed in HBF. Within the subnetwork of these pathways, the gene encoding aspartate aminotransferase (*AAT1*) was shown to be a regulatory hub of these networks. Pharmacological inhibition of this enzyme was shown to perturb biofilm formation, highlighting its potential as a target for biofilm-based infections.

The adaptation of its metabolism is fundamental to the pathogenicity and survival of *C. albicans* within the host [141]. The immune response to *Candida* biofilms is diminished compared to planktonic cells [142], with further evidence suggesting the potential to stimulate biofilm production, resulting in an altered inflammatory output [143]. In response to a device-related *C. albicans* infection, the most responsive leukocyte was shown to be neutrophils [144]. These host cells are able to successfully phagocytose the yeast morphology of *C. albicans*, yet the larger hyphal morphologies require the release of neutrophil extracellular traps (NET) for effective phagocytosis [145]. However, *C. albicans* biofilms are able to inhibit the release of NET during biofilm formation through the production of ECM [146]. This is unlike *C. glabrata* biofilms, whereby a degree of NET are released, although not comparable to NET release against planktonic cells [147]. The differences between these organisms is likely to be the biofilm architecture, predominantly the ECM composition. Despite *Candida* biofilms being more resistant to host defenses, the addition of antifungals has been shown synergise and increase the susceptibility of these communities [148]. This combinatory effect was shown for anidulafungin but not voriconazole, and has also been shown to be active against *C. parapsilosis* biofilms [149]. Furthermore, the addition of the antifungal resulted in increased release of tumor necrosis factor α (TNF-α) compared to untreated biofilms [148]. Interestingly, this same cytokine has been shown to block *C. albicans* biofilm formation through interaction with a major carbohydrate component of the fungal cell wall [150].

The presence of additional environmental stressors such as pH, thermal and oxidative stress, and also the availability of nutrients results in the metabolic adaptation of the biofilm to acclimatize to its surroundings. This, combined with inter-relationships with other yeasts and bacteria, creates multiple permutations of strain specific biofilms, all exhibiting their distinct and unique fingerprints.

## 8. Conclusions and Future Outlook

Extensive studies with laboratory strains have facilitated greater understanding of the biofilm. Underpinning the rationale for these studies, however, is the clinical importance of *Candida* biofilms in human health, and the drive to discover new therapeutic targets. Therefore, reliance on arguably artificial strains to guide our therapeutic search limits our potential. We have presented some ideas supported by recent data to provoke consideration and benefit from working with clinical isolates. Understanding how real strains perform under real clinical conditions can only support and enhance our understanding, whether at a phenotypic or genotypic level. Enhanced or diminished biofilm phenotypes may provide a rationale way to develop new anti-biofilm therapies.

## Figures and Tables

**Table 1:** Mucosal and medical-devices associated *Candida* biofilm infections.

Location	Fungi	Bacteria	Reference
**Mucosal**			
Oral cavity	*C. albicans*, *C. glabrata*	*Streptococcus mutans*, *Streptococcus gordonii*, *Porphomonas gingivalis*, *Staphylococcus aureus*	[27,28,29,30,31]
Respiratory tract	*C. albicans*	*Pseudomonas aeruginosa*	[32,33]
Gastrointestinal tract	*C. albicans*	*Enterococcus faecalis*, *Clostridium difficle*	[34,35]
Vagina	*C. albicans*	*Lactobacillus* spp.	[36]
Wounds	*C. albicans*, *C. auris*	*Pseudomonas aeruginosa*, *Staphylococcus aureus*	[37,38]
**Device-related**			
Denture	*C. albicans*, *C. glabrata*	*Lactobacillus* spp.	[39,40]
Voice prosthesis	*C. albicans*, *C. tropicalis*	*Rothia dentocariosa*	[41,42]
Artificial heart valves	*C. albicans*	*Staphylococcus aureus*, *Staphylococcus epidermidis*	[43,44]
Vascular catheter	*C. albicans*, *C. auris*	*Staphylococcus aureus*, *Staphylococcus epidermidis*	[45,46,47]
Urinary catheter	*C. albicans*, *C. auris*	*Escherichia coli*	[48,49]
